# Role of Dietary Inclusion of Phytobiotics and Mineral Adsorbent Combination on Dairy Cows′ Milk Production, Nutrient Digestibility, Nitrogen Utilization, and Biochemical Parameters

**DOI:** 10.3390/vetsci10030238

**Published:** 2023-03-22

**Authors:** Nikolai P. Buryakov, Larisa V. Sycheva, Vladimir I. Trukhachev, Anastasiya S. Zaikina, Maria A. Buryakova, Ilia N. Nikonov, Alexander S. Petrov, Andrey V. Kravchenko, Mohamed M. Fathala, Ivan K. Medvedev, Dmitrii E. Aleshin

**Affiliations:** 1 Department of Feeding Animals, Institute of Animal Science and Biology, Russian State Agrarian University-Moscow Timiryazev Agricultural Academy, Moscow 125493, Russia; n.buryakov@rgau-msha.ru (N.P.B.); kormlenie@rgau-msha.ru (V.I.T.); azaikina@rgau-msha.ru (A.S.Z.); m.buryakova@rgau-msha.ru (M.A.B.); a.petrov@rgau-msha.ru (A.S.P.); mfathalla@alexu.edu.eg (M.M.F.);; 2 Department of Animal Husbandry, Faculty of Veterinary Medicine and Animal Science, Perm State Agro-Technological University, Perm 614990, Russia; sycheva@pgatu.ru (L.V.S.); kravchenko@pgaru.ru (A.V.K.); 3 Department of Animal Hygiene and Poultry Breeding Named after A.K. Danilova, Faculty of Animal Technologies and Agribusiness, Moscow State Academy of Veterinary Medicine and Biotechnology-MVA Named after K.I. Skryabin, Moscow 109472, Russia; ilnikonov@yandex.ru; 4 Animal Husbandry and Wealth Development Department, Faculty of Veterinary Medicine, Alexandria University, Alexandria 5424041, Egypt

**Keywords:** natural adaptogens, phytobiotic, mineral adsorbents, milk productivity, degradability nutrients, nitrogen utilization, rumen metabolism, biochemical parameters

## Abstract

**Simple Summary:**

The development of dairy cattle breeding is impossible without improving the technologies of raising, feeding, and high-quality breeding work. An important role in the development of cattle milk production is played by local breeds of cows, which have a sufficiently high genetic variability necessary for adaptations to future climate changes, consumer demand, and improvement of economically important traits. Low productivity is a crucial factor in the loss of local livestock breeds, as they are replaced by highly productive foreign cross breeds, which makes many countries, for example, Russia, Serbia, etc., dependent on imported breeds. This study investigated the effect of the inclusion of natural dry grits from *Fucus vesiculosus* and mineral adsorbent from the heat-treated mineral shungite extracted only in Russia in feeding native Suksun breed, and their effect on milk yield, the use of nutrients and nitrogen balance in the diet, and biochemical parameters of the blood.

**Abstract:**

Our research purpose was to study the effect of the inclusion of a combination of phytobiotics in the form of dry *Fucus vesiculosus* grits (FG) and a mineral adsorbent from the heat-treated mineral shungite (TMS) on milk productivity, nutrient digestibility, and biochemical parameters of the Suksun dairy cows. A total of 80 dry-hardy cows of the Suksun breed were divided into four groups (20 heads each), balanced primarily by breed, age, body weight, body condition score, and indicators of milk yield for the previous lactation. The selected cows were with an average live body weight of 512.0 ± 1.28 kg, BCS 3.0–3.5, and parities of 6250 kg milk. The control group (CON) were fed the basic ration only; the second (TMS), third (FG), and fourth (TMS + FG) groups were fed the basic ration provided by 50 g of the mineral adsorbent from heat-treated shungite, 100 g of Fucus grits (*Fucus vesiculosus*), 50 g of the mineral adsorbent from heat-treated shungite, and 100 g of dry grits from *Fucus vesiculosus*, respectively. The total protein content in milk was significantly higher in the group receiving *Fucus vesiculosus* by 0.05% and the group receiving a combination of mineral adsorbent and *Fucus vesiculosus* by 0.03%. The percentage of milk fat content recorded the highest significant value in (TMS) group when compared to the control and represented (4.37 vs. 3.95). The group of cows that received (TMS + FG) revealed a significant difference in the digestibility of both ether extract and crude fiber when compared to the control group and represented (54.74 vs. 51.71 and 60.68 vs. 55.15%), respectively. The cows supplemented with a mineral adsorbent or a combination of mineral adsorbent and *Fucus vesiculosus* revealed a significant difference in the digestibility of ether extract and crude fiber in the group receiving TMS + FG by 3.0% (*p* < 0.05) and 5.5% (*p* < 0.05), respectively. The intake of nitrogen with the diet increased in (FG) and (TMS + FG) groups by 11.3 g (*p* < 0.05) and 13.4 g (*p* < 0.05) of nitrogen. There was an increase (*p* < 0.05) in the concentration of rumen ammonia in the control group compared to the other groups. The glucose content of those cows that received FG and TMS + FG combination increased (*p* < 0.05) by 0.76 mmol/L and 0.90 mmol/l in relation to the control group. The globulin, albumin/globulin ratio, and the level of triglycerides revealed a significant difference between the different experimental groups. In brief, the inclusion of a combination of phytobiotics in the form of dry *Fucus vesiculosus* grits and a mineral adsorbent from the heat-treated mineral shungite in Suksun dairy cows’ diets improved milk composition, digestibility of nutrients, utilization of nitrogen, and did not cause deleterious effects on blood biochemical indicators.

## 1. Introduction

In intensively developing countries, the development of the agro-industrial sector, particularly dairy cattle breeding and production, is one of the priority tasks of development and ensuring the food security of the population [1,2,3]. In addition, the increase in the production and demand for milk and dairy products obtained from cattle in recent decades is due not only to taste characteristics but also to its safety and the way it is obtained [4,5,6]. The raising of local cattle is based primarily on the adaptation of livestock to local habitat conditions, technologies of raising, breeding, feeding, and the use of local feed resources [7,8,9]. All these conditions can vary significantly within large countries (for example, Russian Federation, Kazakhstan, USA, Brazil, Canada, China, Australia, etc.); the reason for this is the peculiarities of the local climate, the type and fertility of soils, average annual temperatures, precipitation, botanical composition of plants, food supply, and individual characteristics of animals raised in different regions [10,11,12]. All these factors directly outline the level and quality of milk, livestock productivity, safety, and chemical composition of the final livestock products, and, accordingly, the level of health of the nation [13].

Sufficient genetic variability of livestock populations is necessary for adaptation to future climate changes and consumer demand and for permanent genetic improvement of economically important traits [7]. Currently, there is a decrease in genetic variability both within and between breeds. Low production is a key factor in the loss of small breeds, as they are replaced by highly productive international cross-border breeds [14,15,16].

In the last two decades, the use of biologically active feed additives, such as organic acids, enzymes, synbiotics, probiotics, prebiotics, phytobiotics and plant extracts, and sorbents and natural stimulants, which can help us obtain environmentally friendly and high-quality agricultural food, has been actively developing in the feeding of livestock, poultry, and aquaculture facilities [17,18].

Algae is a heterogeneous group of plant feeds with complex and often contradictory taxonomy [19]. There are two main types of algae: microalgae (seaweed), which occupy the coastal zone and can be very large, and microalgae, algae of small size, which are found in benthic and coastal habitats, as well as phytoplankton [20,21]. Using algae in animal feeding has a number of advantages in the production of agricultural products; they have the potential to produce protein and energy; they contain a number of biologically active compounds; they can be used as prebiotics [22].

Algae belong to the group of producers with the ability to photosynthesize and are a rich source of organic compounds, including carbohydrates, lipids, proteins, and pigments [23].

In addition to organic sorbents of toxins, one of the alternative products is the use of modern sorbents based on shungite rocks with a particle size of up to 1 mm, which is extracted on an industrial scale from the Zazhoginsky deposit in the Republic of Karelia. In our research, we also used mineral additives based on a shungite substance with a particle size of 0.5–1.5 mm in a heat-treated form [24,25].

However, a combination of such natural phytobiotics as *Fucus vesiculosus* extracted in the White Sea in the northwestern region of Russia and sorbents and mineral additives based on a shungite substance with a particle size of 0.5–1.5 mm in a heat-treated form has not yet been carried out. Consequently, the purpose of our research was to study the effect of a combination of phytobiotics in the form of dry Fucus grits and a mineral adsorbent from the heat-treated mineral shungite on milk yield, nutrient digestibility and biochemical parameters of dairy cows of the Suksun breed of cows.

## 2. Materials and Methods

### 2.1. Ethical Statement

The animal study protocol was approved by the Ethics Committee of the Russian State Agrarian University–Moscow Timiryazev Agricultural Academy (protocol 2022-8 date 6 May 2022).

### 2.2. Animals and Experimental Design

This study was conducted on the Suksun breed of cows of LLC Suksunskoe, located in the village of Sabarka (57°10′34.9032″ 57°12′32.4936″) in the Suksunsky district of Perm Krai, starting from June to December 2022. Eighty dry-hardy cows of the Suksun breed were divided into four groups (20 heads each), balanced primarily by breed, age, body weight, body condition score, and indicators of milk yield for the previous lactation. The selected cows were with an average live body weight of 512.0 ± 1.28 kg, BCS 3.0–3.5, and parities of 6250 kg milk.

The cows were included in the experiment 21 days before the expected calving date when they were fed only 50% of the daily supplementation. Then after calving and throughout the lactation period, supplements were fed in accordance with the dosages illustrated in the study feeding scheme (Table 1) from the first day of lactation to 90 days of the lactation period.

The animals were clinically healthy and kept in the same conditions throughout the experiment. The cows of the first (CON) group were fed according to the basic ration without any additions. The second group (TMS) additionally received 50 g of the mineral adsorbent from heat-treated shungite. The third (FG) group received 100 g of Fucus grits (*Fucus vesiculosus*). The fourth (TMS + FG) group received 50 g of the mineral adsorbent from heat-treated shungite and 100 g of dry grits from *Fucus vesiculosus*).

Throughout the entire duration of the experiment, the animals were subjected to veterinary examination, and later only clinically healthy animals that were in the same conditions of tied confinement took part in the experiment. Cows were fed two times a day and milked twice a day (2X), according to the accepted daily routine on the farm. The inclusion of feed additives was carried out by including them in the composition of concentrates with the recommended dose, which were subsequently included in the composition of the total mixed ration (TMR) and distributed to each group this feed individually in accordance with the accepted feeding regime on the farm. The dry grits *Fucus vesiculosus* phytobiotic and mineral adsorbent (shungite) were fed 21 days before calving with a gradual increase in the dose so that by the beginning of lactation, the animals received the planned amount (50 g of mineral adsorbent from heat-treated shungite and dry grits from *Fucus vesiculosus* in the amount of 100 g per cow per day).

### 2.3. Feed, Animal Feeding, and Dietary Supplement

After calving, the cows were transferred to the department for lactating cows, where they were milked by machine milking twice a day (2X). The daily ration was offered 2 times a day (at 7:00 a.m. and 4:00 p.m.) with unrestricted access to clean water. The diet was balanced in accordance with the recommendations for feeding [26] (Table 2).

### 2.4. Samples Collection and Chemical Analysis

Analysis of the chemical composition and nutritional value of the feed additive of a mineral adsorbent from the shungite rock, which was subjected to heat treatment, and the mineral composition (silicon, zinc, aluminum, iron, manganese, calcium, magnesium, sodium, potassium, sulfur, cobalt, and copper) was determined according to ISO 6869:2000 [27] on a two-beam atomic-absorption spectrophotometer AA-7000 (Shimadzu, Kyoto, Japan). Phosphorus content was determined according to the Interstate standard of the Russian Federation 26657-97 [28] on the spectrophotometer Unico 2100 (United Products & Instruments, Inc., Dayton, NJ, USA).

The analysis of feed analysis was carried out in the Osvoeniya agrozootekhnologij laboratory of the Perm State Agro-Technological University (Perm, Russia). Feed samples were taken in accordance with ISO 6498:2012 [29]. The chemical composition of the products of the physiological experiment was measured by standard methods of the Association of Official Analytical Chemists [30,31]: dry matter (DM, AOCS 930.15); ether extract (EE, 920.85; AOAC); crude ash (CA, AOCS 942.05); nitrogen content and crude protein (CP, AOCS 976.05); crude fiber (CF, AOCS Ba-05 standard procedure); calcium according to the national standard of Russian Federation 26570-95 [32]; phosphorus according to the national standard of Russian Federation 26657-97 [28]. Neutral-detergent fiber (NDF) determined by the method Van Soest et al. (1991) [33], accepted for use in fiber analyzer ANKOM A2000 (ANKOM TM Technology, Macedon, NY, USA).

### 2.5. Milk Sampling and Analysis

Milk yield was measured weekly individually from each animal by receiving milk into individual recorded tanks, while during the balance experiment was measured daily. Cows were milked twice a day (2X) at 05:00 and 17:00. Actual daily milk yield, actual total (total) milk yield, daily milk yield of 4% fat corrected milk (4% FCM), total milk yield of 4% FCM, mass fraction of protein, and fat was measured during the entire lactation period (90 DIM). Milk samples were collected (from all dairy cows under the study) in individual containers and stored in the refrigerator at a temperature of +4 °C before being sent to the laboratory.

For collecting a representative sample of milk, a part of an individual milk yield immediately after milking cows was taken into a special tank with a sampler and poured into a certain container and thoroughly mixed. Representative samples of milk volume were taken during two successive milking sessions (morning and evening) so that the volume of the sample from the milk of one milking is set in proportion to the proportion of this milking in the daily milk yield. The analysis of the chemical composition of milk was carried out on the CombiFoss FT+ analyzer (Foss Electric, Hillered, Denmark) for qualitative characteristics (protein and fat). All samples were measured twice. Daily and total milk yields of natural milk and milk with a 4% adjusted fat content (FCM) were calculated based on the results of control milking and the fat content in milk and calculated according to the formula developed by Kugenev et al. (1988) [34].

### 2.6. Digestibility of Nutrients and the Use of Nitrogen in the Diet

The digestibility and use of nutrients in the diets were established based on the results of balance experiments conducted at 3 months of lactation in accordance with methodological recommendations from the All-Russia Institute of Animal Husbandry (2016) [26]. During the experiment, 20 cows (5 heads from each group) were selected, which were homogeneous in milk productivity and reflected the average value for the group, and were placed in an individual stall without walking for 5 days. On the 90th day of lactation, representative samples (milk, feces, and urine) were taken daily for five consecutive days (balance experiment) to study the chemical composition, digestibility, and average daily nitrogen balance.

To prevent microbial degradation and loss of volatile nitrogen in the form of NH_3_, a 10% hydrochloric acid solution and toluene were added to the feces and urine at the rate of 10% HCl and 0.5% toluene by the weight of the average sample. All the fecal matter was collected in barrels behind each animal. Urine and feces were weighed daily 2 times a day in the morning after morning feeding and in the evening, as well as filling. Fecal discharge was recorded in the same way as urine discharge, with each event collected in a bucket by a technician with weight and time registration. Every morning during the N-balance study, the feces of each animal were mixed, and 200 g samples of feces and urine were collected for subsequent chemical analysis.

The analysis of the chemical composition of the litter was carried out similarly to the methods presented in Section 2.4.

Apparent digestibility coefficients (*DC*, %) of nutrients in the diet were evaluated using Equation (1):

(1) $$DC=\frac{intake\;nutrient-excreted\;in\;feces}{intake\;nutrient}\times 100\%$$

### 2.7. Blood Sampling and Analysis

During the first 30 DIM and at the 90th DIM, after conducting a balance experiment to determine the digestibility of nutrients and the use of nitrogen in the diet, blood samples were taken for further studying of morphological and biochemical parameters. Blood samples were taken by puncture of the jugular vein at 10:00 a.m. into blood collection tubes (Zhejiang Gongdong Medical Technology Co., Ltd., Huangyan, China) containing a coagulation activator (silicon oxide) to study the biochemical parameters of serum. Blood was taken from cows 3 h after morning feeding, after a balancing experiment, to avoid the effects of stress on animals during blood collection. After clot formation (10–15 min), the samples were centrifuged at 3000× *g* for 15 min at 4 °C to extract the serum. Similarly, vials with blood anticoagulant K2-EDTA (Zhejiang Gongdong Medical Technology Co., Ltd., Huangyan, China) were selected to obtain whole blood and study the morphological parameters of the blood. The obtained samples were placed in a portable isothermal bag with cold storage batteries and stored at a temperature of +4 °C until delivery to the laboratory approximately 1 h after sampling. The study of biochemical blood parameters for 1 month (30 DIM) and 3 months (90 DIM) of lactation of cows and morphological parameters for only 1 month (30 DIM) was carried out in laboratory Osvoeniya agrozootekhnologij Perm State Agro-Technological University (Perm, Russia).

Morphological parameters of blood were determined on a VetScan HM5 hematological veterinary analyzer (serial number: 366820, Abaxis, Inc., Union City, CA, USA) using VetScan HM5 reagent kits (Abaxis, Inc., USA). Biochemical parameters of blood were determined using the Beckman Coulter AU 480 analyzer (Beckman Coulter. Inc., Brea, CA, USA). The cyanocobalamin (vitamin B_12_) content was determined by the immuno-chemiluminescent method on ACCESS 2 analyzers (Beckman Coulter Inc., USA); Infinite F50 (Tecan Austria, GmbH, Grödig, Austria); Lazurite (Dynex Technologies Inc., Chantilly, VA, USA) using BioRad Lyphochek Immunoassay Plus level 3 control materials (Bio-Rad, Hercules, CA, USA).

### 2.8. Fermentation Indicators in the Rumen

Rumen fluid samples were taken during the same period (90 DIM), as well as blood samples of the same animals after conducting an experiment to determine the digestibility and use of nitrogen in the diet in order to study physico-biochemical parameters. Samples of ruminal fluid were collected on day 2 from the end of the balance experiment to determine the metabolism (pH, NH_3_, and short-chain fatty acids) in the rumen. Samples of ruminal fluid were taken from all animals 3 h after the morning feeding using a rumen probe according to the method of Larsen et al. [35] and Buryakov et al. [36]. The ruminal fluid was collected in a sterile container with a volume of 0.5 L and divided into several samples: the first for the analysis of ammonia and the total amount of volatile fatty acids; the second for determining the molar ratio of different types of short-chain fatty acids. The first portion (200 mL) was drained into a bucket and was not used for subsequent analysis. The obtained samples were placed in a portable isothermal bag with cold storage batteries and stored at a temperature of +4 °C during delivery to the chemical and analytical laboratory. After delivery to the laboratory, the contents of the ruminal fluid were filtered through four layers of gauze.

The sample of filtered ruminal fluid was used to determine the total amount of short-chain fatty acids (SCFAs) by steam distillation using a Markham device according to the method described in Buryakov et al. [36]. Ammonia was determined using the Conway microdiffusion method based on the displacement of ammonia from ammonium salts with a concentrated alkaline solution followed by absorption with a solution of sulfuric acid.

### 2.9. Statistical Analysis

Before carrying out the statistical analysis, all data were tested for normality and homogeneity by Shapiro–Wilk’s and Levene’s tests, respectively. Beforehand processing percentile data, an arcsine transformation was used. Data were statistically analyzed using the statistical analysis program SPSS [37]. One-way ANOVA followed by Tukey’s multiple comparison tests (post hoc test) were used to check the significance and compare the experimental groups [38], according to the following statistical model:

Xijk = μ + Ai + eijk

where

Xik = An individual observation;

μ = Overall mean;

Ai = Effect of ith treatment;

eik = Random error.

## 3. Results

### 3.1. Analysis of Nutritional Value and Chemical Composition of Additives

The analysis of the mineral content in shungite was carried out several times before the start of the study and during the entire study. The results obtained (average) are presented in Table 3.

Rendering to our research, the mineral rock shungite contains total carbon—34.8% mass and mineral compounds (in the form of oxides, % mass): silicon (54.89), aluminum (3.67), iron (2.43), manganese (<0.02), calcium (0.19), magnesium (1.07), sodium (<0.3), potassium (1.05), and phosphorus (0.06). In terms of the element content, a high content of iron (929.46 mg/kg), calcium (369.5 mg/kg), potassium (78.87 mg/k), magnesium (7.16 mg/kg), copper (7.02 mg/kg), and zinc (29.20 mg/kg) was noted.

### 3.2. Milk Production and Quality Indicators

In Table 4, the results of lactation indicators of the Suksun cows breed are given.

The results obtained on the dairy productivity of the Suksun dairy breed confirm that the inclusion of feed additives in the form of heat-treated shungite and *Fucus vesiculosus*, both individually and together, does not adversely affect the dairy productivity of cows. In some indicators, it reveals that the content of productivity in the experimental groups (with additives) is higher than in the control. The total protein content (mass fraction in %) in cow milk was higher (*p* < 0.05) in the group receiving *Fucus vesiculosus* by 0.05% (*p* < 0.05) and the group receiving TMS + FG by 0.03% (*p* < 0.05), respectively. Moreover, it was noted that the percentage of milk fat content recorded a significant increase between the supplemented groups and nonsupplemented ones and represented (4.37, 4.35, 4.66 vs. 3.95) for (TMS, FG, TMS + FG, and control group, respectively.

### 3.3. Indicators of Digestibility and Nitrogen Balance in Cows

Means ± standard errors for the digestibility coefficient of nutrients in lactating cows are presented in Table 5.

According to the data obtained, the introduction of dry grits of *Fucus vesiculosus* and heat-treated shungite into the diet both separately (TMS and FG) and together (TMS + FG) did not significantly negatively affect the digestibility of DM, OM, CP, CF, and NFE. On the other hand, the introduction of TMS and FG feed additives into the diets of lactating cows revealed a significant difference in the digestibility of the other nutrients as (EE and CF), which increased in the group receiving TMS + FG by 3.0% (*p* < 0.05) and 5.5% (*p* < 0.05), respectively. Results of nitrogen metabolism in lactating cows are presented in Table 6.

It was noted that the intake of nitrogen with the diet increased in the third and fourth groups by 11.3 g (*p* < 0.05) and 13.4 g (*p* < 0.05) of nitrogen, respectively. The analysis of nitrogen utilization showed that the introduction of feed additives did not have a significant decrease in the efficiency of nitrogen use. However, it can be seen from the current results that nitrogen use (retention) was 3.71%, 7.10%, and 9.35% in the TMS, FG, and TMS + FG groups, higher than the level in the control group.

### 3.4. Rumen Fermentation Indicators

The effect of the inclusion of feed additives on the concentration of pH, ammonia, the total amount of volatile fatty acids, and the dry matter content of bacteria and dry matter content protozoa is presented in Table 7.

The concentration of ammonia in the ruminal fluid in all groups was within the normal range. However, there was a slight increase in the concentration of ammonia in the control group compared to the other groups, and the differences were significant.

### 3.5. Blood Biochemical Parameters

The results of the biochemical blood analysis of the Suksun dairy breed are presented in Table 8 and Table 9.

Analysis of biochemical and morphological parameters of the blood of a Suksun breed cow at the beginning of lactation (30 DIM) did not reveal significant changes. However, it should be noted that the value of all indicators was within the reference values for dairy cows.

According to the results represented in Table 9, the inclusion of an adsorbent based on heat-treated mineral rock shungite and dry grits (*Fucus vesiculosus*), extracted in the White Sea in the northwestern region of Russia, both separately and together, did not have a negative effect on the biochemical parameters of blood serum. The glucose content of those who received FG and TMS + FG as part of the diets increased (*p* < 0.05) by 0.76 mmol/L and 0.90 mmol/L in relation to the control group.

With reference to the globulin and albumin/globulin ratio revealed a significant difference between the different experimental groups. Moreover, the level of triglycerides was significantly decreased between the groups and represented 0.22, 0.17, 0.15, and 0.22 mmol/L for TMS, FG, TMS + FG, and the control group, respectively.

## 4. Discussion

### 4.1. Analysis of Nutritional Value and Chemical Composition of Additives

The mineral adsorbent based on the rock shungite is of particular interest for investigation because it is a resource in the USA (Dead River Basin, Marquette Co., New Hampshire, MI, USA) [39], Austria (four deposit districts: Tyrol; Salzburg; Styria) [40], Democratic Republic of the Congo (Kambove District, Upper Katanga)b [41], India (Kadapa District, Rayalasima Region, Andhra Pradesh) [42], Ukraine (Mlynkovskoye iron ore district, Krivoi Rog-Kremenchug zone, Ukrainian shield) [43], Republic of Kazakhstan (Almaty region) [44], and Russia (Republic of Karelia) [45,46,47], where it occurs in a large volume. It is known that there are quite a lot of ways to use it in various sectors of the national economy, such as creating water filters, used as an antioxidant and anti-inflammatory agent in veterinary medicine, and as a sorbent and feed additive. However, a very small number of works are devoted [46,47,48,49,50,51].

Polunina et al. [52] studied the influence of thermo- and mechano-chemical modification of the mineral shungite from Karelia (Russian) on its chemical composition and physicochemical properties of their effect on the sorption of toxins and phenols, while the sorption properties of shungite from the Koksu deposit (Kazakhstan) were studied by Ongarbayev et al. [44]. Thus, in the works of Charykova et al. [47], it is shown that the material from the shungite deposit of Zazhoginskoe (Republic of Karelia) contains 30% carbon and 70% silicon. Shungite (type III) contains iron, nickel, copper, and zinc, mainly in the form of sulfides and oxides. In our studies, a similar carbon content was noted in the amount of total carbon (34.8% mass) and mineral compounds (in the form of oxides, % mass): silicon (54.89), aluminum (3.67), iron (2.43), manganese (<0.02), calcium (0.19), magnesium (1.07), sodium (<0.3), potassium (1.05), and phosphorus (0.06), which probably can be regarded as this feed not only as a sorbent but also a source of minerals of natural origin.

### 4.2. The Milk Productivity and Quality Indicators

In recent decades, the increased use of natural and synthetic sorbents of toxic substances and mycotoxins has become widely used in modern animal production sectors [18,53]. In our studies, it was found that the inclusion of the mineral shungite did not have a significant effect on milk yield (*p* > 0.05), which is consistent with the results of Khachlouf et al. (2018) [54]; they studied the effect of a mineral sorbent from zeolites in feed for lactating cows. As a result of their study, it was found that moderate levels of zeolite (200–400 g per cow per day) had increased milk yield, but the content of milk fat and protein, as well as DMI, was not affected by zeolite [55]. In our studies, an increase in the content of total milk protein in the group with FG was noted, which indicates a more efficient use of ammonia in the rumen of these cows.

Milk fat is the most volatile and energy-intensive component of milk, and it is easily affected by nutrition, followed by protein and lactose, respectively, which remain relatively stable [56,57,58]. The rich chemical composition and fatty acid composition of seaweed have a high content of long-chain polyunsaturated fatty acids docosahexaenoic acid and eicosapentaenoic acid [59].

The composition of fatty acids of *Fucus vesiculosus* probably contributed to the detection of the intake into the small intestine of cows together with the mineral sorbent in a related form. Some authors noted that mineral shungite could sorb some inorganic and organic acids [60,61]. In our opinion, when feeding the cows in the fourth group (TMS + FG), the sorbent partially sorbs these acids and passes into the small intestine, where during digestion, the medium has an alkaline reaction and possibly sorption of part of organic acids occurs, which subsequently become an integral part of cow’s milk fat. However, this process, when using these feed additives in combination, is still insufficiently studied in the feeding of ruminants.

### 4.3. Indicators of Digestibility and Nitrogen Balance in Cows

Due to its chemical composition and the content of polyunsaturated fatty acids, seaweed can serve as a raw material for the production of feed and feed additives [62]. Studies [59] stated a decrease in feed intake due to the addition of algae during the first few weeks due to the specific smell of algae.

The presence of an effective feedback mechanism to reduce or slow down the intake of the additive is also assumed in relation to the regulation of the osmolality of the animal’s rumen. A study of the use of algae in feeding cattle showed a decrease in feed consumption and, accordingly, productivity. However, in our case, algae in dried form were used directly in feeding adult animals. So, probably, in our case, the more attractive taste of algae served as an incentive to feed consumption and also showed that the content of a high amount of biologically active substances affects the digestibility of nutrients. In our studies, significant digestibility of nutrients such as EE was noted in the group receiving both algae and the shungite, which is probably due to the more efficient synthesis of fibrolytic enzymes from the vitamin B_12_ content in algae. When feeding algae in its pure form, it did not have a significant effect on the digestibility of CF; however, its use in combination was significantly increased when used in combination with the mineral supplement shungite.

Our research revealed a significant increase in the digestibility coefficients of ether extract and crude fiber, as well as nitrogen intake from diets in dairy cows receiving a mixture of sorbent based on shungite and brown algae grits (TMS + FG) as part of the diets. The optimal ratio of the sorbent that sorted chemicals that negatively affect the process (excess ammonia) of rumen digestion and digestibility probably had an optimal effect. Thus, similar results were obtained by Leupp et al. [63] on steers fed with low-quality hay, in which a positive effect on the consumption of dry matter was found when feeding brown seaweed flour. Similarly to the authors’ conclusions, feeding seaweed extract (*Gracilaria* sp.) did not affect DM consumption in lactating cows when feeding 10 g and 20 g of seaweed extract per day [64].

### 4.4. Rumen Fermentation Indicators

In our studies, there were no significant differences between the groups in terms of ruminal contents, with the exception of ammonia content; there was a significant decrease in its level in the groups receiving the mineral shungite in its pure form and mixed with algae grits in the diet. Moreover, we did not find significant differences in pH or SCFAs concentration, which contradicts the results obtained in the study by [65].

In the studies of [24,25,45,47,48], it was noted that shungite has sorption to inorganic substances (heavy metals, dioxins, pesticide radicals, chlorine, benzene, etc.) and organic pollutants, such as ammonia, surfactants, and petroleum products. In our opinion, a decrease in ammonia content could probably occur in the rumen for three reasons. Firstly, there was more intensive absorption of ammonia by the walls of the rumen and its entry into the blood. However, according to our studies in all experimental groups (except for the fourth), the urea content in the blood was lower than in the control group. Secondly, it is probably due to the sorption properties of shungite and, finally, to the intensive synthesis of microbial protein.

According to this statement, one of the directions of the use of the mineral shungite is not only the adsorption of toxic substances but also excess ammonia formed during the decomposition of feed and diets with a high proportion of protein and concentrate breakdown. We obtained similar results when assessing the ammonia content in the fourth group (TMS + FG); however, the ammonia content was slightly higher compared to the second group (TMS), but no significant differences were found between them. At the same time, the introduction of a mixture of TMS and FG did not have a highly intensive reduction in ammonia levels, probably due to the formation of an additional source of ammonia from algae proteins.

### 4.5. Blood Biochemical Parameters

In our studies, the inclusion of feed additives in the first month did not show significant differences in blood biochemical parameters. However, significant values in blood parameters were noted at 3 months of lactation. At the beginning of the experiment, the inclusion of a TMS + FG in the northwestern region of Russia, both separately and together, did not have a significant effect on morphological and biochemical parameters at the beginning of feeding (30 DIM) between experimental groups except for the total protein contents, which revealed significant increase between the different groups.

In our study, when sampling blood, it was found that the introduction of feed additives in the diet did not have a negative effect on morphological and biochemical parameters. The glucose content (90 DIM) of those who received FG and TMS + FG as part of the diets increased by 0.81 mmol/L and 0.90 mmol/L in relation to the control group. With reference to the globulin and albumin/globulin ratio revealed a significant difference between the different experimental groups. Moreover, the level of triglycerides was significantly decreased between the groups and represented 0.22, 0.17, 0.15, and 0.22 mmol/L for TMS, FG, TMS + FG, and the control group, respectively.

Probably due to the fact that brown algae contain a high amount of minerals, including trace elements and organic substances—biologically active and prebiotic substances [66]—they affect the synthesis of volatile fatty acids (propionic, butyric, etc.). Similar results in reducing blood glucose when feeding brown algae were noted in the studies [67,68]. It was reported that the increase in these parameters is the result of increased hematopoiesis and improved assimilation of the algae supplement due to the individual content of folic acid and vitamin B_12_ in it.

Lipids, including triglycerides, cholesterol, and phospholipids, as well as their derivatives, provide the body with energy and play a significant role in the functioning of the endocrine system and some intracellular signaling pathways [67,68,69]. The decrease in triglycerides is due to the content of certain carbohydrates in Fucus algae (fucoidan and alginic acid) on lipid metabolism in animals and humans. Similar results were established in another study [68] when feeding fucoidan isolated from the brown algae Fucus evanesces to mice with dyslipidemia; a significant decrease in the level of lipid metabolism (cholesterol and triglycerides) was observed. Similar results were obtained in the work of [69] when using fucoidan preparations; it helps to reduce the content of triglycerides and restore the ratio of lipid fractions included in plasma lipoproteins, as well as the content of polyunsaturated fatty acids in brown algae promotes the synthesis of phospholipid fractions from triglycerides and reduces the proportion of cholesterol in membranes, promotes its replacement with phospholipid fractions, in particular phosphatidylcholine and phosphatidylethanolamine, as well as metabolically active fractions, that allows them to be used as a means to restore the structure of cell membranes in case of metabolic disorders.

## 5. Conclusions

Based on the aforementioned results, we can conclude that the inclusion of a mineral adsorbent from heat-treated shungite in an amount of 50 g and dry grits from *Fucus vesiculosus* in an amount of 100 g in the diet of the Suksun dairy breed at the first stage of lactation improved the protein content in milk, ammonia concentration, digestibility of ether extract and crude fiber, nitrogen utilization, and did not cause deleterious effects on the general health of animals.

## Figures and Tables

**Table 1:** Feeding scheme and research design.

Groups	Cows Number (*n*)	The Program of Feeding Cows
CON	20	Basic diet, without inclusion of mineral adsorbent (shungite) and dry grits *Fucus vesiculosus*.
TMS	20	BD + mineral adsorbent from heat-treated shungite (Zazhoginsky mine, Republic of Karelia, Russian Federation) in the amount of 50 g.
FG	20	BD + dry grits from *Fucus vesiculosus* (Obtained from the White Sea, Republic of Karelia, Russian Federation) in the amount of 100 g.
TMS + FG	20	BD + mineral adsorbent from heat-treated shungite in the amount 50 g and dry grits from *Fucus vesiculosus* in the amount of 100 g

**Table 2:** Feed content in the ration and nutritional value of the diet lactating cows.

Feeds of Diet	Content (kg)	Feeds of Diet	Content (kg)
Wheat straw	2.0	Rapeseed cake	1.5
Cereal-legume hay	4.0	Flattened wheat	1.8
Cereal-legume silage	13.0	Flattened barley	1.8
Clover haylage	16.0	Vitamin-trace mineral premixes	0.05
**Nutritional value**			
Metabolic energy (ME) MJ	175.4	Phosphorus, g	89.0
Dry matter (DM), kg	17.2	Magnesium, g	40.0
ME per 1 kg DM, MJ/kg	10.2	Potassium, g	151.0
Crude protein, g	2572.0	Sodium, g	46.0
Digestible protein, g	1542.0	Sulfur, g	40.0
Degradable protein, g	1786.0	Iron, mg	2711.0
Ungradable protein, g	981.0	Copper, mg	173.0
Neutral detergent fiber, g	6422.0	Zinc, mg	1148.0
Acid detergent fiber, g	3911.0	Manganese, mg	1125.0
Crude fiber, g	3427.0	Cobalt, mg	14.3
Starch, g	2440.0	Iodine, mg	15.8
Sugars, g	627.0	Carotene, mg	803.0
Essential extract, g	536.0	Vitamin D, thousands of IU	17.8
Calcium, g	133.0	Vitamin E, mg	1457.0

**Table 3:** The content of minerals in the rock shungite mined in the Republic of Karelia (Russia).

Trace Mineral Element	Concentration, mg/kg	Trace Mineral Element	Concentration, mg/kg
Sodium	68.05 ± 5.80	Copper	6.72 ± 1.91
Magnesium	157.58 ± 28.54	Zinc	39.34 ± 15.67
Aluminum	133.15 ± 46.12	Bromine	4.73 ± 0.24
Potassium	75.10 ± 20.42	Selenium	0.67 ± 0.10
Calcium	378.87 ± 26.28	Rubidium	0.59 ± 0.19
Titan	1.18 ± 0.06	Strontium	8.79 ± 0.60
Vanadium	1.43 ± 0.43	Zirconium	0.70 ± 0.22
Chrome	2.95 ± 0.97	Molybdenum	0.78 ± 0.10
Manganese	6.24 ± 1.62	Iodine	2.99 ± 0.45
Iron	903.49 ± 26.17	Barium	16.49 ± 2.64
Cobalt	0.60 ± 0.11	Tungsten	0.45 ± 0.11
Nickel	9.19 ± 2.82	Lead	8.05 ± 1.35

**Table 4:** Milk productivity and quality indicators of the Suksun cows breed (90 DIM).

Parameters	Type of Feed Additive in the Diet				*p*-Value
	CON	TMS	FG	TMS + FG	
Milk yield, kg	1808.17 ± 44.01	1845.40 ± 71.02	1863.50 ± 62.55	1863.50 ± 62.55	0.904
Average daily milk yield, kg	20.09 ± 0.49	20.50 ± 0.79	20.71 ± 0.69	20.71 ± 0.69	0.904
FCM yield, kg	1793.61 ± 48.11	1834.01 ± 74.69	1863.07 ± 60.71	1863.07 ± 60.71	0.836
Average daily of FCM yield, kg	19.93 ± 0.53	20.38 ± 0.83	20.70 ± 0.67	20.70 ± 0.67	0.837
Milk fat content, %	3.95 ± 0.03 ^b^	4.37 ± 0.13 ^a^	4.35 ± 0.19 ^a^	4.36 ± 0.05 ^a^	0.018
Milk total protein content, %	3.03 ± 0.01 ^b^	3.03 ± 0.01 ^b^	3.08 ± 0.02 ^a^	3.06 ± 0.01 ^ab^	0.012
GFC, kg	70.63 ± 2.03	72.30 ± 3.08	73.76 ± 2.36	73.76 ± 2.36	0.784
GPC, kg	55.90 ± 1.85	55.90 ± 1.85	57.71 ± 1.47	56.66 ± 0.86	0.825

Values are expressed as means ± standard error. Means denoted within the same row with different superscripts are significant (*p* < 0.05). GFC—total amount of pure fat. GPC—total amount of pure protein. FCM—milk with a 4% adjusted fat content. CON—control group. TMS—mineral adsorbent from heat-treated shungite in the amount of 50 g. FG—dry grits from *Fucus vesiculosus* in the amount of 100 g. TMS + FG—mineral adsorbent from heat-treated shungite in the amount of 50 g and dry grifts from *Fucus vesiculosus* in the amount of 100 g.

**Table 5:** Indicators of digestibility of nutrients in lactating dairy cows.

Parameters, %	Type of Feed Additive in the Diet				*p*-Value
	CON	TMS	FG	TMS + FG	
DM	67.86 ± 0.27	68.54 ± 0.29	68.86 ± 0.46	69.31 ± 0.56	0.170
OM	68.74 ± 0.23	69.71 ± 0.39	70.36 ± 0.82	70.52 ± 0.38	0.131
CP	70.88 ± 0.68	72.04 ± 0.20	70.82 ± 1.20	72.32 ± 1.18	0.566
EE	51.71 ± 0.37 ^b^	52.38 ± 0.47 ^ab^	50.75 ± 0.87 ^b^	54.74 ± 0.72 ^a^	0.012
CF	55.15 ± 0.90 ^b^	57.23 ± 1.34 ^ab^	55.62 ± 1.36 ^b^	60.68 ± 0.58 ^a^	0.027
NFE	74.22 ± 0.30	75.33 ± 0.66	74.44 ± 0.44	76.12 ± 0.64	0.118

CON—control group. TMS—mineral adsorbent from heat-treated shungite in the amount of 50 g. FG—dry grits from *Fucus vesiculosus* in the amount of 100 g. TMS + FG—mineral adsorbent from heat-treated shungite in the amount of 50 g and dry grifts from *Fucus vesiculosus* in the amount of 100 g. Values are expressed as means ± SE. Means denoted with different superscripts within the same row are significant (*p* < 0.05). DM—dry matter, OM—organic matter, EE—essential extract, CP—crude protein, CF—crude fiber, and NFE—nitrogen-free extractive substances.

**Table 6:** The utilization of nitrogen by dairy cows of the Suksun breed.

Parameters	Type of Feed Additive in the Diet				*p*-Value
	CON	TMS	FG	TMS + FG	
Consumed nitrogen, g	349.58 ± 2.10 ^b^	355.04 ± 1.21 ^ab^	360.88 ± 1.40 ^a^	363.02 ± 2.21 ^a^	0.003
Excreted with feces, g	101.91 ± 1.63	99.69 ± 0.28	105.26 ± 3.98	104.07 ± 0.49	0.337
Digestible nitrogen, g	247.82 ± 3.84	255.35 ± 1.05	255.62 ± 5.26	258.92 ± 2.41	0.227
Excreted with milk, g	92.70 ± 6.42	95.94 ± 5.86	98.87 ± 3.50	100.95 ± 3.05	0.673
Excreted in urine, g	153.41 ± 8.13	157.23 ± 5.34	160.04 ± 2.76	155.19 ± 3.62	0.838
Assimilable nitrogen, g	94.41 ± 6.09	98.12 ± 6.05	101.12 ± 3.36	103.76 ± 3.35	0.592
Absorbed from consumed, %	27.01 ± 1.77	27.63 ± 1.65	28.02 ± 0.82	28.58 ± 0.86	0.866
Absorbed from digested, %	38.14 ± 2.67	38.41 ± 2.25	39.55 ± 0.75	40.08 ± 1.25	0.868
Balance (±), g	+1.71 ± 0.35	+2.18 ± 0.19	+2.24 ± 0.14	+2.81 ± 0.34	0.108

CON—control group. TMS—mineral adsorbent from heat-treated shungite in the amount of 50 g. FG—dry grits from *Fucus vesiculosus* in the amount of 100 g. TMS + FG—mineral adsorbent from heat-treated shungite in the amount of 50 g and dry grifts from *Fucus vesiculosus* in the amount of 100 g. Values are expressed as means ± SE. Means denoted with different superscripts within the same row are significant (*p* < 0.05).

**Table 7:** Indicators of fermentation in the rumen of dairy cows of the Suksun breed.

Parameters	Type of Feed Additive in the Diet				*p*-Value
	CON	TMS	FG	TMS + FG	
pH	6.47 ± 0.08	6.38 ± 0.06	6.68 ± 0.30	6.44 ± 0.06	0.526
Ammonia, mg%	10.21 ± 0.57 ^a^	8.01 ± 0.21 ^b^	9.26 ± 0.15 ^ab^	8.09 ± 0.14 ^b^	0.004
SCFAs, mmol/100 mL	7.96 ± 0.77	8.28 ± 0.76	8.89 ± 0.43	8.59 ± 0.34	0.740
DM of protozoa, g/100 mL	0.19 ± 0.02	0.21 ± 0.04	0.16 ± 0.06	0.17 ± 0.05	0.870
DM of bacteria, g/100 mL	0.20 ± 0.01	0.21 ± 0.02	0.20 ± 0.02	0.21 ± 0.01	0.934

CON—control group. TMS—mineral adsorbent from heat-treated shungite in the amount of 50 g. FG—dry grits from *Fucus vesiculosus* in the amount of 100 g. TMS + FG—mineral adsorbent from heat-treated shungite in the amount of 50 g and dry grifts from *Fucus vesiculosus* in the amount of 100 g. Values are expressed as means ± SE. Means denoted with different superscripts within the same row are significant (*p* < 0.05).

**Table 8:** Blood biochemical parameters of Suksun dairy breed (30 DIM).

Parameters	Type of Feed Additive in the Diet				*p*-Value
	CON	TMS	FG	TMS + FG	
Leucocytes, 10^9^/L	6.17 ± 0.37	7.72 ± 0.56	6.02 ± 0.43	6.72 ± 0.37	0.091
Erythrocytes, 10^12^/L	6.95 ± 0.40	6.30 ± 0.27	5.29 ± 1.00	7.53 ± 0.09	0.095
Hemoglobin, g/L	109.00 ± 3.06	97.00 ± 4.16	104.00 ± 4.16	107.67 ± 3.84	0.197
Hematocrit, %	40.33 ± 1.54	36.21 ± 1.78	37.77 ± 1.68	39.35 ± 1.77	0.391
MCV, fl	58.00 ± 2.65	57.33 ± 1.76	58.33 ± 3.18	52.33 ± 1.45	0.309
MCH, pg	15.77 ± 0.90	15.40 ± 0.38	15.60 ± 0.95	14.37 ± 0.35	0.527
MCHC, g/dl	27.07 ± 0.42	26.83 ± 0.19	26.73 ± 0.17	27.47 ± 0.24	0.301
Platelets, 10^9^/L	334.33 ± 72.47	316.67 ± 31.69	315.33 ± 50.96	375.67 ± 63.62	0.862
Neutrophils, %	26.67 ± 10.94	33.93 ± 4.14	36.00 ± 3.35	37.53 ± 6.35	0.698
Lymphocytes, %	63.27 ± 9.16	57.50 ± 5.31	54.13 ± 5.43	53.77 ± 7.12	0.755
Monocytes, %	6.20 ± 3.50	3.90 ± 1.99	2.87 ± 1.30	1.80 ± 0.75	0.471
Eosinophils, %	3.43 ± 0.48	3.30 ± 0.85	5.93 ± 1.66	5.40 ± 0.46	0.216
Basophils, %	1.33 ± 0.17	1.37 ± 0.13	1.07 ± 0.43	1.50 ± 0.06	0.658
Glucose, mmol/L	4.24 ± 0.17	4.70 ± 0.11	4.53 ± 0.50	4.56 ± 0.76	0.914
Creatinine, µmol/L	148.33 ± 11.89	144.67 ± 14.89	144.67 ± 5.55	145.33 ± 23.84	0.998
Urea, mmol/L	3.63 ± 0.19	3.53 ± 0.23	3.57 ± 0.27	4.27 ± 0.20	0.144
Total bilirubin, µmol/L	3.47 ± 1.19	5.15 ± 0.26	3.28 ± 0.37	4.67 ± 0.89	0.319
Cholesterol, mmol/L	3.06 ± 0.16	4.40 ± 1.00	3.97 ± 0.24	4.13 ± 0.30	0.389
Triglycerides, mmol/L	0.16 ± 0.02	0.21 ± 0.05	0.14 ± 0.02	0.18 ± 0.04	0.554
Calcium, mmol/L	2.19 ± 0.06	2.48 ± 0.18	2.26 ± 0.04	2.24 ± 0.08	0.292
Phosphorus, mmol/L	1.19 ± 0.09	1.22 ± 0.09	1.23 ± 0.08	1.31 ± 0.07	0.750
Aspartate aminotransferase, U/L	34.97 ± 4.64	41.40 ± 2.95	39.17 ± 6.77	42.97 ± 5.10	0.709
Alanine aminotransferase, U/L	79.00 ± 6.66	64.00 ± 2.31	78.00 ± 3.46	77.33 ± 4.81	0.146
Gamma-glutamyltransferase, U/L	23.30 ± 0.15	22.80 ± 1.30	25.37 ± 1.56	23.03 ± 0.85	0.389
Alkaline phosphatase, U/L	52.30 ± 1.42	51.50 ± 1.27	52.00 ± 0.87	52.47 ± 1.28	0.946

CON—control group. TMS—mineral adsorbent from heat-treated shungite in the amount of 50 g. FG—dry grits from *Fucus vesiculosus* in the amount of 100 g. TMS + FG—mineral adsorbent from heat-treated shungite in the amount of 50 g and dry grifts from *Fucus vesiculosus* in the amount of 100 g. Values are expressed as means ± SE. MCHC—mean concentration of hemoglobin per erythrocyte; MCV—mean corpuscular volume; MCH—mean cell hemoglobin.

**Table 9:** Blood biochemical parameters of Suksun dairy breed (90 DIM).

Parameters	Type of Feed Additive in the Diet				*p*-Value
	CON	TMS	FG	TMS + FG	
Glucose, mmol/L	2.37 ± 0.18 ^b^	2.47 ± 0.12 ^b^	3.13 ± 0.09 ^a^	3.27 ± 0.09 ^a^	0.002
Creatinine, µmol/L	73.00 ± 4.04	85.33 ± 4.41	77.67 ± 6.01	87.67 ± 9.17	0.376
Total protein, g/L	78.23 ± 1.62	79.50 ± 4.04	83.47 ± 1.34	82.70 ± 0.35	0.369
Urea, mmol/L	7.07 ± 0.62	6.93 ± 0.23	6.83 ± 0.24	6.77 ± 0.29	0.948
Albumin, g/L	32.60 ± 1.95	35.47 ± 1.04	35.00 ± 0.26	35.63 ± 0.45	0.277
Globulin, g/L	45.63 ± 2.38 ^a^	34.03 ± 1.00 ^b^	38.47 ± 1.12 ^ab^	40.40 ± 3.65 ^ab^	0.043
Albumin/globulin	0.72 ± 0.07 ^b^	1.04 ± 0.02 ^a^	0.91 ± 0.02 ^ab^	0.89 ± 0.08 ^ab^	0.025
Urea/Creatinine ratio	98.31 ± 14.13	81.98 ± 7.16	89.07 ± 7.51	79.28 ± 10.24	0.577
Total bilirubin, µmol/L	4.00 ± 0.15	3.93 ± 0.27	4.30 ± 0.42	3.57 ± 0.12	0.348
Bilirubin straight, µmol/L	0.53 ± 0.12	0.43 ± 0.15	0.53 ± 0.15	0.33 ± 0.03	0.613
Cholesterol, mmol/L	5.23 ± 0.09	5.20 ± 0.12	5.00 ± 0.10	5.10 ± 0.15	0.520
Triglycerides, mmol/L	0.22 ± 0.02 ^a^	0.22 ± 0.02 ^a^	0.15 ± 0.01 ^b^	0.17 ± 0.01 ^ab^	0.019
Calcium, mmol/L	2.37 ± 0.03	2.40 ± 0.06	2.43 ± 0.03	2.40 ± 0.06	0.802
Phosphorus, mmol/L	1.70 ± 0.12	1.70 ± 0.06	1.72 ± 0.06	1.72 ± 0.02	0.995
Magnesium, mmol/L	0.93 ± 0.03	1.00 ± 0.06	0.97 ± 0.03	1.13 ± 0.07	0.091
Potassium, mmol/L	3.87 ± 0.13	4.03 ± 0.18	3.80 ± 0.17	4.13 ± 0.28	0.644
Sodium, mmol/L	138.33 ± 0.67	139.33 ± 1.20	138.00 ± 1.53	139.33 ± 1.20	0.802
Chlorine, mmol/L	93.00 ± 1.00	96.67 ± 2.67	95.00 ± 3.51	96.67 ± 1.20	0.659
Iron, mmol/L	23.03 ± 1.95	24.23 ± 0.92	21.67 ± 1.00	23.70 ± 2.55	0.754
Acidity, unit pH	7.75 ± 0.03	7.68 ± 0.09	7.72 ± 0.03	7.76 ± 0.01	0.633
Aspartate aminotransferase, U/L	70.33 ± 6.49	84.33 ± 8.69	91.67 ± 11.41	103.33 ± 17.95	0.328
Alanine aminotransferase, U/L	38.67 ± 1.76	42.67 ± 1.45	43.67 ± 0.67	43.33 ± 2.67	0.248
Gamma-glutamyltransferase, U/L	27.07 ± 3.86	17.97 ± 0.45	25.33 ± 2.89	29.43 ± 6.42	0.283
Alpha-amylase, U/L	112.67 ± 5.81	87.00 ± 11.15	108.00 ± 7.37	112.00 ± 1.53	0.113
Alkaline phosphatase, U/L	53.67 ± 0.33	55.33 ± 2.85	56.00 ± 2.08	53.67 ± 2.73	0.837
Creatine kinase, U/L	113.00 ± 14.93	133.67 ± 37.95	166.33 ± 44.01	162.33 ± 63.55	0.803
Lactate dehydrogenase, U/L	963.33 ± 101.14	1134.00 ± 100.18	1152.67 ± 160.84	1364.00 ± 50.90	0.164
Cyanocobalamin (vitamin B_12_), pg/mL	82.67 ± 9.21	86.00 ± 14.29	106.00 ± 21.38	168.67 ± 74.93	0.440

CON—control group. TMS—mineral adsorbent from heat-treated shungite in the amount of 50 g. FG—dry grits from *Fucus vesiculosus* in the amount of 100 g. TMS + FG—mineral adsorbent from heat-treated shungite in the amount of 50 g and dry grifts from *Fucus vesiculosus* in the amount of 100 g. Values are expressed as means ± SE. Means denoted within the same row with different superscripts are significant (*p* < 0.05).

## Data Availability

The raw data supporting the conclusions of this article will be made available by the authors without undue reservation. The data presented in this study are available upon request from the corresponding author.
